# Analysis of Compromising Video Disturbances through Power Line

**DOI:** 10.3390/s22010267

**Published:** 2021-12-30

**Authors:** Bogdan Trip, Vlad Butnariu, Mădălin Vizitiu, Alexandru Boitan, Simona Halunga

**Affiliations:** 1 Special Telecommunications Service, 060044 Bucharest, Romania; bogdan.trip@stsnet.ro (B.T.); vlad.butnariu@stsnet.ro (V.B.); alexandru.vizitiu@stsnet.ro (M.V.); 2 Telecommunications & Information Technology Faculty, Telecommunications Department, University Politehnica of Bucharest, Electronics, 060042 Bucharest, Romania

**Keywords:** TEMPEST, recovery, propagation, distance, compromising, video, protection, unintended, measurement, signal to noise ratio

## Abstract

In this article, we present results on research performed in the TEMPEST domain, which studies the electromagnetic disturbances generated unintentionally by electronic equipment as well as the methods to protect the information processed by this equipment against these electromagnetic phenomena. The highest vulnerability of information leakage is attributed to the display video signal from the TEMPEST domain perspective. Examples of far-range propagation on a power line of this type of disturbance will be illustrated for the first time. Thus, the examples will highlight the possibility of recovering processed information at distances of 1, 10 and 50 m. There are published articles studying electromagnetic disturbances generated by electronic equipment propagating on power cables of such equipment but no studies on their long-distance propagation. Our research aims to raise awareness in the scientific community and the general public of the existence of such vulnerabilities that can compromise confidential or sensitive information that can make the difference between success or failure in the business sector, for example, or can harm personal privacy, which is also important for us all. Countermeasures to reduce or even eliminate these threats will also be presented based on the analysis of the signal-to noise-ratio recorded during our research.

## 1. Introduction

We live in an age of continuous digitization and thus we are all inseparable from our smart phones, multifunction tablets, and portable personal computers (laptops), and all these information technology and communications (IT&C) gadgets have become an extension of our personality. It is no longer necessary to memorize the telephone numbers of our acquaintances, the addresses of holiday destinations, mathematical and physical formulas, conversion or transformation formulas and we do not even perform simple arithmetic calculations because we have an Internet connection and IT&C applications that solve all these problems. When we leave for the long-awaited holidays, we no longer have paper maps spread all over the car; we enter the destination in a navigation application, and we are guided visually and acoustically along the entire route.

In addition, as a result of continuous digitization, in the next 5 years in Romania, we will reach interaction with public institutions remotely without requiring a physical presence to obtain permits, certificates or any other official documents or, for example, to register a vehicle, issue an identity card or any document, or perform various automation processes remotely, as is already the case in European countries that are at a higher stage in the digitization process.

Since the emergence of the COVID-19 pandemic throughout the world and due to restrictions imposed to limit the spread of the SARS-COV-2 virus such as physical distancing, limiting community and global circulation, and conducting on-line school classes, remote communication has continually overtaken physical communication. Companies have also been forced to organise on-line business meetings through various videoconferencing applications and have allowed their employees to work from home using Virtual Private Network (VPN) connections to their data centres. Therefore, we have been forced over the last two years to become more attached to our electronic gadgets. This electronic equipment comes with display screens that help us to interact with the equipment in question, either by simply viewing the information displayed or by transmitting multi-touch commands, as is the case of display screens with touch capability (which can be resistive or capacitive).

It is a well-known fact that any electronic equipment generates electromagnetic emissions in the surrounding space or electromagnetic disturbances in the power lines to which it is connected or on the data lines that are connected to it. The electromagnetic compatibility (EMC) domain studies both the ability of electronic equipment to operate in an environment without disturbing the proper functioning of other equipment found in the proximity of the targeted equipment (EMI—ElectroMagnetic Interference) and the immunity of the equipment to external electromagnetic interference (EMS—ElectroMagnetic Susceptibility). The European Committee for Electrotechnical Standardization established general limits for such disturbances as well as standardized methods for operating conditions [1]; the International Electrotechnical Commission specifies the required characteristics and performances for the equipment used for measuring those disturbances [2] as well as the requirements for maximum levels of such radio disturbances and standardized operating conditions [3]. The United States Department of Defence also established verification requirements necessary to control electromagnetic interference emissions for the equipment used for military purposes [4].

The TEMPEST domain, however, studies [5,6,7,8] those electromagnetic emissions or disturbances generated by the targeted electronic equipment that can propagate and radiate both in the surrounding space and that can conduct on data or power lines (which are similar to the EMC domain thus far) that contain, in some form, the information manipulated by the monitored equipment. The EMC domain refers to all these electromagnetic emissions as electromagnetic interference, while the TEMPEST domain refers to them as compromising electromagnetic emissions or emanations (CE—compromising emissions or emanations). The difference is that only a part of the electromagnetic interferences studied by the EMC domain can be identified with the compromising emissions studied by the TEMPEST domain because not all electromagnetic emissions generated by electronic equipment contain, in some way, the information processed by it. The TEMPEST domain actually studies the vulnerabilities of electronic equipment to reveal the information processed by generating these CE emissions because it is possible to partially or totally restore the processed information by applying filters and signal processing on it.

In this research, the electromagnetic disturbances generated by two display devices injected onto the power supply line to which they are connected will be analysed, as well as the possibility of their far-range propagation on the power line.

The possibility of recovering the information displayed was first reported by Wim Van Eck in 1985 [9]. This article was the first warning sent to the scientific community in which the author presented relevant results of electromagnetic infiltration using a TV receiver, a dipole antenna and a video synchronization circuit. The impact was strong because the equipment used was common, cheap and accessible. Other crucial research has been developed by Marcus Kuhn, who studied CE electromagnetic disturbances for both cathode ray tube (CRT) [10] and liquid crystal display (LCD) [11,12]. He is the only researcher who has attempted to justify, to some extent, the values of the TEMPEST standard limits [7,8], starting from the limits of the EMC standard [1,3]. This task has been difficult since those critical CE signal levels have not been documented in the past and because those limits have a maximum classification level and cannot be disclosed to the general public. Rafal Przesmycki and his collaborators studied the compromising electromagnetic radiation generated by Digital Visual Interface (DVI) [13] and High Definition Multimedia video Interface (HDMI) [14] on the basis of research conducted at the Polish Military Communications Institute. In the same research centre, Ireneusz Kubiak investigated the influence of display brightness on the image recovery capabilities from CE electromagnetic radiation of Video Graphics Array (VGA) and DVI interfaces [15]. He also proposed an innovative method to counteract these security vulnerabilities by using security fonts [16] for personal computers (PCs) that can be successfully applied even to printing devices [17]. The effectiveness of the security fonts developed by Kubiak (symmetric and asymmetric fonts) and which have obtained Polish Office Pattern protection in the form of industrial design no. 24487 (2018) and patent no. 231691 (2019), has also been analysed in [18]. The effectiveness of the security provided by these fonts in the case of the use of display colours was also studied in [16,19,20]. In [21], electromagnetic jamming equipment is proposed as an alternative TEMPEST countermeasure for the compromising emissions radiated into free space by electronic equipment. The proposed jamming equipment, named TEMPEST guard, generates a customized jamming according to the pixel clock of the equipment display, thus reducing the vulnerability of the equipment to electromagnetic infiltration processes. The authors of [22] presented the confidentiality threats related to remote communication devices, such as monitors, personal computers and Voice over Internet Protocol (VoIP) terminals and a large number of results, focused both on video and audio information. Reference [23] presented a new type of attack, named screen glaring, that allows reading the information displayed on a mobile device based on an antenna and a software-defined-radio (SDR) device and how this attack can be addressed using a deep learning classifier. The authors of [24] presented a method to automatically identify the display type based on the reconstructed image intercepted from electromagnetic radiation using support vector machines (SVM), and the performances of the proposed solution were evaluated. In [25], a new class of optical Tempest attacks were identified, which recovers the sound based on the interception of the emanations generated by the power indicator LED of different devices such as speakers, USB hub splitters, and microcontrollers, and the efficiency of those attacks are tested in different scenarios.

This research is structured into six sections as follows. Section 2 presents the measurement equipment as well as the test bed. Section 3 presents the pre-detection of CE emissions, which is a helpful tool in specialized TEMPEST measurements that are similar to measurements performed in the EMC field. The major difference with the EMC domain is precisely that in TEMPEST we do not stop at this step only by comparing the frequency sweeps with the standard limits, we must also investigate the limit exceedances, whether they are caused by the investigated CE signals or not, and we also have to carry out an additional level assessment of these compromising emissions. Section 4 shows the recorded signal-to-noise ratio (SNR) reports linked to the video recovery for CE reception at the specified distances. Section 5 presents some low-cost solutions to counteract the reported phenomena, while Section 6 briefly presents the conclusions of our research.

## 2. Measurement Test Bed

**Figure 1:**
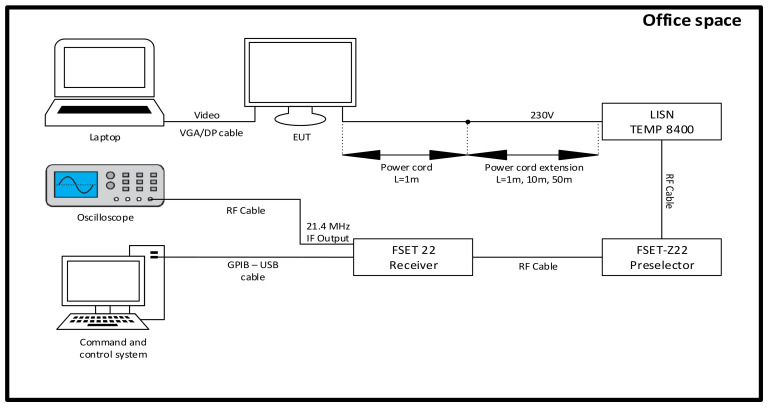
Measurement test bed.

All measurements were performed in an office space environment, as long-distance propagation cannot be tested in a specialised laboratory that is not generously sized, on the order of tens of metres. A laptop computer was chosen as the exciter, to which a commercial AOC monitor was connected via a DisplayPort (DP) cable and a commercial HP monitor via a VGA cable, one at a time. The power line disturbance analysis for each monitor was performed using a Line Impedance Stabilization Network, LISN TEMP 8400 electromagnetic transducer [26], operating in the 9 kHz–1 GHz range. LISNs are used to measure conducted emissions on power lines. The LISN draws power from the ordinary wall outlet and supplies it to the equipment under test (EUT). LISN type equipment performs a number of important functions: they provide stable line impedance, prevent external noise coupling and ensure proper connection to measurement equipment over 50-ohm impedance. The primary function of LISN equipment is to provide stable, normalised impedance on the power line. This function is important because the impedance of the power line through a standard wall outlet can vary greatly, depending on how and where the wiring is connected. The amount of noise generated by the EUT present at the LISN measurement port is directly related to the impedance of the supply line versus the impedance of the EUT. These two impedances effectively create a voltage divider network for the EUT noise, allowing only a fraction of the noise voltage to reach the measurement port. Therefore, the accuracy and precision of the measurements depend on the LISN impedance over the full range of measurement frequencies. The second important function of a LISN is to isolate external noise that may be present on the power line. The input stage of the measurement receiver is sensitive and susceptible to damage. Thus, the third function of the LISN equipment is the most important and allows the low-level RF noise from the EUT to be easily coupled to the input of the measurement receiver. The LISN TEMP 8400 is a symmetrical alternative current (AC) network that consists of two identical channels for the lines “A” and “B” especially constructed in order to avoid intermodulation interference. The manufacturers strongly recommend that when connecting this equipment to any receiver, we should take into consideration that lower frequency interferences can cause damage to the measuring receiver; thus, we must use either a high pass filter for protection, or, at least, a direct current (DC) blocker to eliminate the DC component of the signal. Another important point to know is that the LISN TEMP 8400 can withstand a maximum continuous current consumption of 16 Amps for the EUT, or for a short period of time (3 min at most) 25 Amps. The EUT is linked via a Schuko adapter (TEMP 8401 DE-N) that is connected to the A and B ports of the LISN, thus powering the EUT and enabling the operator to analyze and measure the electromagnetic interferences present on the power line of the equipment, using the “Output A” and “Output B” N-female measurement ports. The measurement test bed is presented in Figure 1.

The reception of the signal captured by the electromagnetic transducer will be provided by a system consisting of a TEMPEST wideband FSET22 receiver [27] and a pre-selector FSET Z22 which are controlled by specialized TEMPEST control software. The FSET 22 test receiver can be called a TEMPEST receiver mostly because of its maximum resolution bandwidth (RBW) capability, of up to 500 MHz, which is an important parameter, ensuring that broadband signals are fully detected and made available for further processing at the intermediate frequency (IF) or video outputs. It has a frequency range of 100 Hz up to 22 GHz, which is enough to cover the spectrum of interest for the TEMPEST domain. It is important to highlight that this domain mostly focuses on low level signals and this receiver can provide an internal noise level down to -140 dBm, which translates into a higher sensitivity of the receiver. The receiver operates linked with a FSET-Z22 preselector which filters—by using numerous high-pass, low-pass and band-pass filters of extremely low insertion loss that suppress strong signals improving the sensitivity of the entire receiving system, and amplifies, using switchable 10/20/30 dB preamplifiers for maximum sensitivity—the signal of interest.

In the pre-detection phase, the monitors will be connected to the LISN using only their AC power cable. We will consider this configuration as “0 m” propagation since the EUT is powered directly from the LISN without using an extension cable. We will continue the tests by connecting the EUT to extension cables with lengths of 1, 10 and 50 m and performing SNR and image recovery measurements for each case.

An extension cable with reel was used to analyse the signal propagation at a distance of 50 m. In this case, two series of tests were developed: a test when the cable was wrapped around the roller and a test when the cable was unwound and stretched over a distance of 50 m such that electromagnetic induction was minimal. There are disadvantages to both situations: in the case of using the cable unwound, it can pick up electromagnetic interference from the environment, as the 50 m power cable is unscreened and is the closest situation to everyday reality while in the case of using the cable tight over the coil, electromagnetic interference is induced along the power cable from one loop to the other.

## 3. Pre-Detection Phase

### 3.1. Frequency Domain Sweeps

In order to identify the frequencies on which the compromising signal occurs, first, we performed several frequency sweeps, in accordance with the video parameters of the targeted displays. Despite the fact that the AOC display has been used with a VGA cable, while the HP display has been connected via a DisplayPort cable, both EUTs have a maximum resolution of 1920 × 1080 pixels with a screen refresh rate of 60 Hz.

The HP monitor used in our tests is an EliteDisplay E243 monitor, product number HSTND-9581, and was produced in 2019. The manufacturer states that it is a 2017 model and assures us that it meets the following EMC standards: EN 55032:2012 Class B, EN 55024:2010, EN 61000-3-2:2014, EN 61000-3-3:2013 and FCC CFR 47 Part 15.

The AOC monitor conversely is model AOC I2269VWM, product number 215LM00040, and was produced in 2017. The manufacturer states that it is a model from 2014 and assures us that it meets the following EMC standards: EN60950-1:2006 and A11+A1, EN 55022:2006+A1: 2007, EN55024:1998+A1:2001+A2:2003, EN 61000-3-2:2006+A1: 2009+A2: 2009, EN 61000-3-3:2008, 2006/95/EC, 2004/108/EC and 2005/32/EC.

These display devices are commercial equipment and this means that they do not have additional EMI filters installed on the equipment power line as would be the case with equivalent TEMPEST (shielded) equipment.

**Figure 2:**
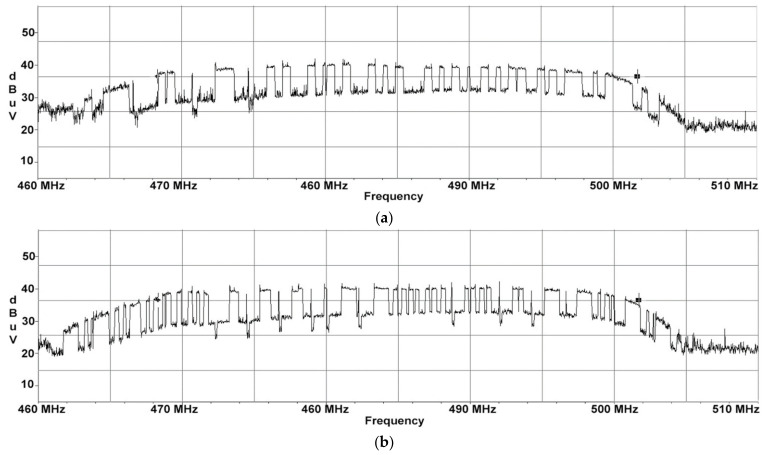
AOC display unit: (**a**) frequency sweeps performed for test signal consisting of 3 horizontal bars equal in width; (**b**) frequency sweeps performed for test signal consisting of 1 thick horizontal line and 3 thin horizontal lines equal in width.

For the AOC display unit, we performed frequency sweeps in the range 460÷510 MHz which are illustrated in Figure 2. These sweeps were carried out in order to emphasize the presence of the unwanted CE and to show the differences between the two cases where the display was showing each test message at a time.

**Figure 3:**
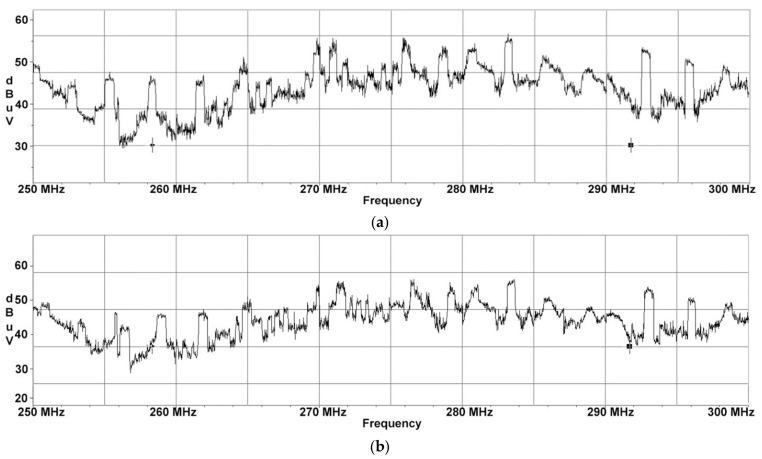
HP display unit: (**a**) frequency sweeps performed for test signal consisting of 3 horizontal bars equal in width; (**b**) frequency sweeps performed for test signal consisting of 1 thick horizontal line and 3 thin horizontal lines equal in width.

Frequency sweeps were performed with two different test signals (displayed images): one test signal consisting of three horizontal lines equal in width and the second consisting of one thick horizontal line and three thin horizontal lines equal in width. The test images were chosen for easy identification in the electromagnetic spectrum. When a static image is displayed for a certain period, the display video signal is periodic during this time interval. Corresponding to the 60 Hz screen refresh rate is a period of 16.6 milliseconds (ms), which means that a minimum sweep time to be utilized on the receiver should be twice the period of one video frame. In our case, we chose a sweep time of 50 ms. Furthermore, due to the fact that both EUTs will display images composed of horizontally thin white stripes on a black background that would occupy a narrower spectrum than in the case of an image containing alternating black and white pixels, during the pre-detection phase we concluded that a resolution bandwidth of 10 MHz would be more than enough to capture the compromising signal. The binary appearance (of 0 and 1) of the received electromagnetic spectrum (generated by the equipment under test) is not due to a synchronization between the display and the receiver but to the receiver’s set parameters, respectively the receive filter (RBW) and the sweep time. The RBW chosen must allow the reception of the first lobe of the spectrum corresponding to the monitored signal and the sweep time must be set to a minimum of one period (best two periods) of the electrical signal generating the electromagnetic radiation. The most important role in this sense is played by the test images. The display video signal contains the video information of each line transmitted successively until the last line of the video frame. Video frames are separated by Vertical Sync signals while successive video lines are separated by Horizontal Sync. After transmission of the last line of the current video frame, the first line of the next video frame is transmitted next. In the case of HP display, sweeps in the range of 250 to 300 MHz have been performed, which are illustrated in Figure 3.

Although this EUT generates electromagnetic disturbances with a maximum SNR in another frequency range, the two EUT have almost similar electromagnetic footprints, which is caused by the fact that they operate with similar parameters (refresh rate and display resolution) but using different interfaces.

### 3.2. Time Domain Analysis

As a further verification of the presence of the compromising signal on the frequencies identified during the sweep procedure, an oscilloscope has been linked to the receiver via the 21.4 MHz intermediate frequency output in order to measure the timing of the video frame of both EUTs. In this step the same test images were used as in the frequency sweep procedure section.

The oscilloscope is set to capture a 50 ms time Span, which means that it will always display three full video frames for analysis. The span parameter specifies the range between the start and stop frequencies for a spectrum analyzer or time window set for an oscilloscope. In the case of the AOC monitor, for analysis of the compromising signal in the time domain, the receiver was tuned on 484 MHz central frequency.

**Figure 4:**
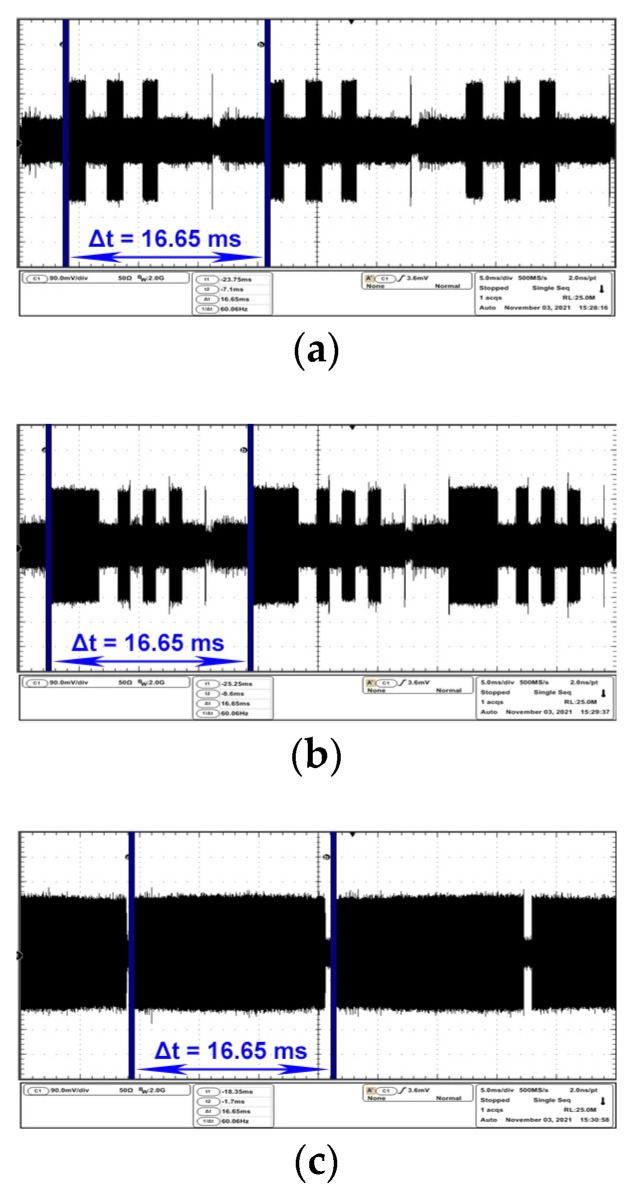
AOC display: (**a**) frame period corresponding to an image containing three white equal stripes on black background; (**b**) frame period corresponding to an image containing one thick white stripe and three thin white equal stripes on black background; (**c**) frame period corresponding to a white image.

Figure 4a,b contains the waveform represented in the time domain corresponding to the moment when the AOC monitor displays white horizontal bars on a black background, while Figure 4c represents the result after displaying a completely white image on the EUT. The maximum level of compromising radiation may vary according to the colours contained in the test message, according to the frequency on which the compromising signal propagates and according to the equipment on which the information in question is processed. In this case, the maximum radiation level is given by the colour white, while the minimum radiation level is given by the colour black.

The electromagnetic disturbances reported here are permanently generated by the EUT and have a sequential character which is given by the characteristics of the video display signal.

**Figure 5:**
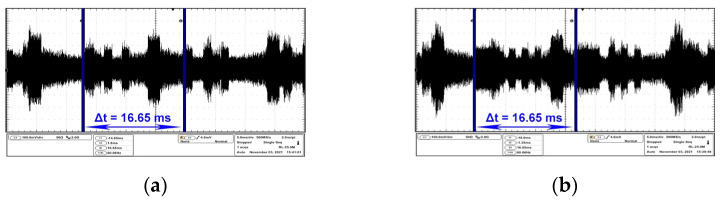
HP display: (**a**) frame period corresponding to an image containing three black equal stripes on white background; (**b**) frame period corresponding to an image containing one thick black stripe and three thin black equal stripes on black background.

In all the above figures, the repetition period of the video frame is measured as 16.65 ms, resulting in a monitor refresh period of 60 Hz. The HP monitor was analyzed on the central frequency of 274 MHz. Figure 5a,b emphasizes the time domain parameters of the video frames while on the EUT two types of black bars are displayed on a white background, contrary to the examples mentioned in Figure 4a–c for the AOC monitor.

HP monitor analysis also involves some impediments due to the fact that the perturbances generated by the EUT’s AC power supply unit propagate on this frequency, which can lead to measurement errors or uncertainty when investigating the compromising emissions of the equipment.

**Figure 6:**
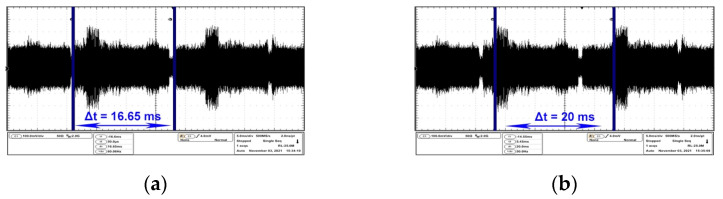
HP display: (**a**) frame period of 16.6 ms corresponding to an image containing a black image; (**b**) time interval between two emissions generated by the EUT’s AC power source measured at 20 ms.

Basically, Figure 6a,b differs only in the positioning of the markers. Thus, in Figure 6a, the markers indicate the period of the video frame, while in Figure 6b, the period of the electromagnetic noise is indicated.

This electromagnetic noise co-exists with the video signal CE on the EUT power line. In Figure 6a,b the most favourable case is shown: the target signal level is minimal when showing a complete black image, thus giving the possibility to visualize and measure only the electromagnetic noise generated by the power supply network.

## 4. SNR vs. Image Recovery

This section presents the results of a two-step experiment: first, the SNR of the compromising signal was measured while each monitor displayed a simple test message consisting of three horizontal bars for distances of 0, 1, 10 and 50 m, and second, the image reconstruction was performed for each EUT at all distances mentioned above. We investigated the SNR instead of absolute level of CE signals because we considered that the visibility of these signals in the analysed spectrum is more important. Since, for this experiment, we will be looking to perform SNR measurements instead of level measurements for both monitors at various propagation distances through the power line, it was decided to use the Average detector over the Max Peak detector available on the receiver for a smoother view of the target signal versus noise.

**Figure 7:**
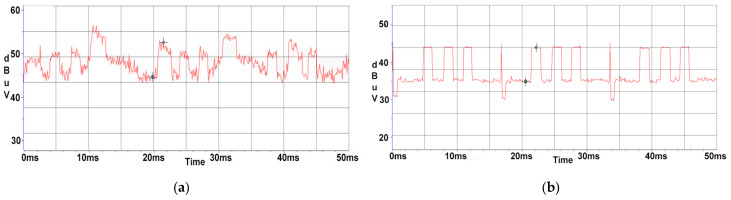
AOC display: (**a**) displayed waveform using a Max Peak detector; (**b**) displayed waveform using an Average detector.

Due to the fact that the signals targeted in our research are narrower than the electromagnetic noise generated by the EUT’s power supply unit, a measurement with a peak detector can completely mask the lower-level narrower signal because the detector responds predominantly to the peaks of the broadband signal. Average detection, conversely, is used to suppress broadband signals and is therefore well suited to recover the amplitudes of narrowband signals. It should be noted that the recovery of CE signals in the presence of electromagnetic noise is the primary purpose of using the average detector. On the 274 MHz central frequency, the HP monitor also contains noise due to its power supply, which could compromise the measurements accuracy, as shown in Figure 7a,b.

**Figure 8:**
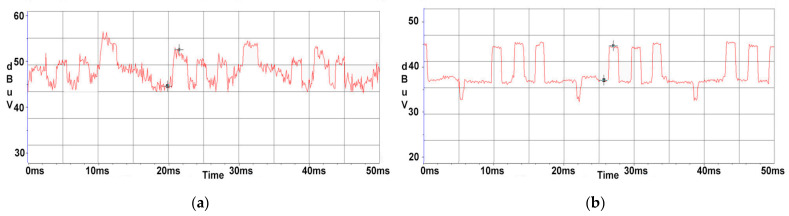
HP display: (**a**) displayed waveform using a Max Peak detector; (**b**) displayed waveform using an Average detector.

In the case of the AOC monitor, the differences are not considerable, due to the fact that there are no additional perturbances propagated through the power line at this central frequency. On the 274 MHz frequency, the HP monitor also contains noise due to its power supply mains, which could be an impediment to measurements, as shown in Figure 8a. In this situation, it is observed in Figure 8b that by using an Average Detector, the SNR significantly improves in means that the 50 Hz signal is diminished to a comparable to noise floor value.

### 4.1. AOC Display

As concluded in the predetection phase it is expected that the recovered image from the AOC display unit should be slightly better qualitatively than the one from the HP display unit due to the lack of additional high amplitude parasitic signals, as it can be observed in Figure 8.

**Figure 9:**
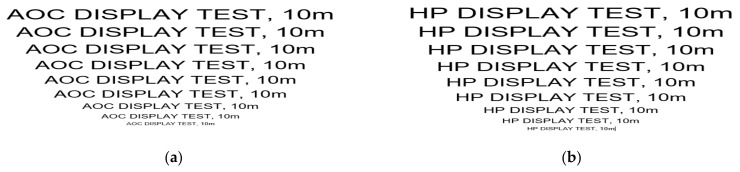
Examples of test images: (**a**) for AOC display; (**b**) for HP display.

As a further part of our experiment, we created video test images containing text with the EUT producer’s name, followed by the distance of the power cord interposed throughout our measurement chain using decreasing different font sizes of 72, 48, 36, 28, 26, 20, 18 and 12. We have also illustrated in Figure 9 two examples of test images in order to make an easy comparison between the test images used in our tests and the images recovered by receiving CE signals. In order to verify the existence of the CE emissions generated by the tested equipment and which are propagated on their power supply line, a specialized TEMPEST software application was used, which has also implemented the raster function.

The raster module allows the serialized display of the reconstructed image from the samples corresponding to the IF signal provided by the test receiver. This is possible if the time parameters of the analyzed video signal are known (horizontal and vertical synchronizations—video sync).

However, we are not allowed to disclose clear and detailed specifications regarding this specialized software application for the TEMPEST domain. Unfortunately, when we talk about the TEMPEST domain, we often find an impenetrable wall that consists of classified information that cannot be published. This is the biggest difficulty that needs to be overcome by researchers who want to publish research in this technical field and that forces us to pay more attention to the information contained in published articles and make sure that we do not insert classified information in published articles which may have criminal consequences according to the national legislation in force.

**Figure 10:**
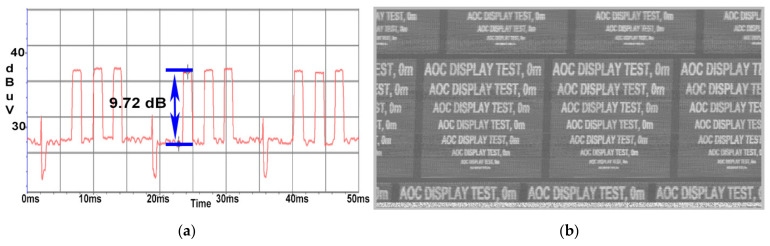
AOC display: (**a**) SNR measured at a distance of 0 m; (**b**) image recovered at a distance of 0 m.

In the case of the AOC display, as expected, a high SNR value of 9.7 dB was obtained, as shown in Figure 10a; and image reconstruction was achieved as illustrated in Figure 10b due to the fact that the AOC display unit was connected directly with the power cable to the LISN equipment and that no additional attenuation of our signal of interest along the measurement chain was involved.

**Figure 11:**
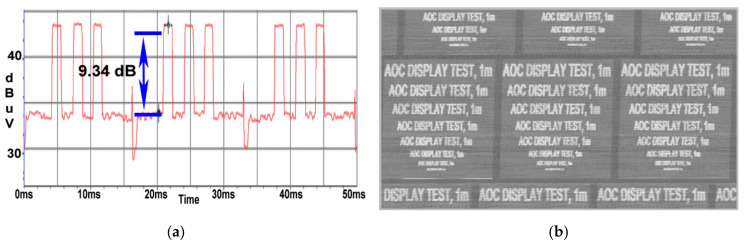
AOC display: (**a**) SNR measured at a distance of 1m; (**b**) image recovered at a distance of 1 m.

By inserting a 1 m long extension cable between the power cable of the EUT and the LISN, the CE value compared to the background noise is still high, as can be seen in Figure 11a, at about 9.3 dB, while the image was recovered at a quality level comparable to that shown in Figure 11b. In this scenario, the signal is not distorted in a way that would affect either the SNR measurement or the image reconstruction.

**Figure 12:**
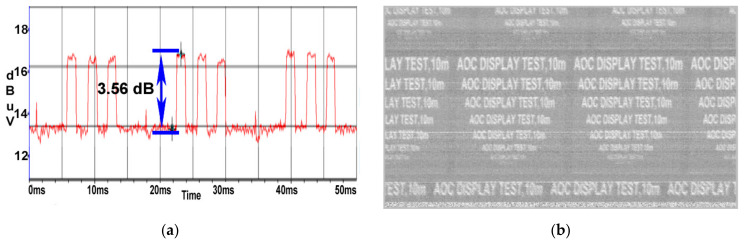
AOC display: (**a**) SNR measured at a distance of 10 m; (**b**) image recovered at a distance of 10 m.

At a distance of 10 m, the SNR value drops to 3.5 dB as illustrated in Figure 12a; thus, the quality of the image retrieved from the AOC display unit degraded, the test message being readable until a 26 font is used as displayed in Figure 12b.

**Figure 13:**
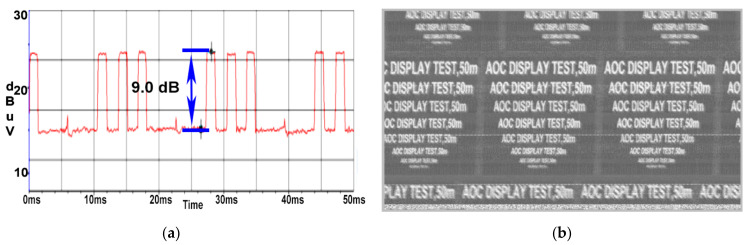
AOC display: (**a**) SNR measured at a distance of 50 m when the cable reel was wound; (**b**) image recovered at a distance of 50 m when the cable reel was wound.

Although the cable reel of 50 m was utilised, the fact that the power cord was wound and each spire would radiate electromagnetic field around it thus inducing CE from coil to coil, the targeted signal would be received with a high amplitude, resulting in a high SNR value of 9.0 dB illustrated in Figure 13a, and the information from the reconstructed image would be intelligible down to a font of 22, as can be seen in Figure 13b.

**Figure 14:**
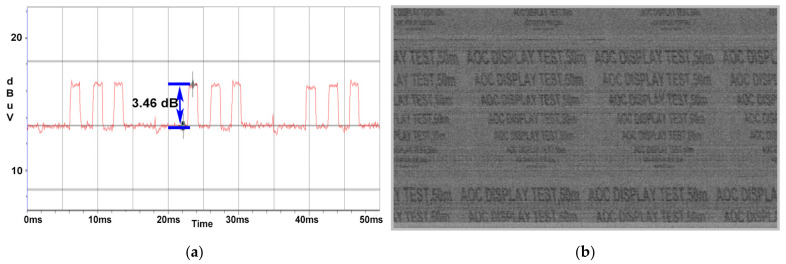
AOC display: (**a**) SNR measured at a distance of 50 m; (**b**) image recovered at a distance of 50 m.

When we unwound the cable from the 50 m reel, the effects are visible, leading to a smaller SNR value, of 3.1 dB, as highlighted in Figure 14a and a poor quality of the reconstructed image as shown in Figure 14b.

The value of the SNR of 3.5 dB, measured by using a 10 m power cord as shown in Figure 11a, is comparable with the value obtained in this stage of the experiment, but the rasterised image has a reduced quality and is less intelligible; thus, the 0.4 dB difference in SNR value may be noticeable for the video image restoration process.

### 4.2. HP Display

The image recovery facility was ensured by a Digital Signal Processor (DSP) connected to the 21.4 MHz IF output of the FSET 22 receiver, which means that the Average detector does not improve the quality of the image obtained during the video recovery process as the detector only affects the receiver display and not the signal provided on the IF output.

**Figure 15:**
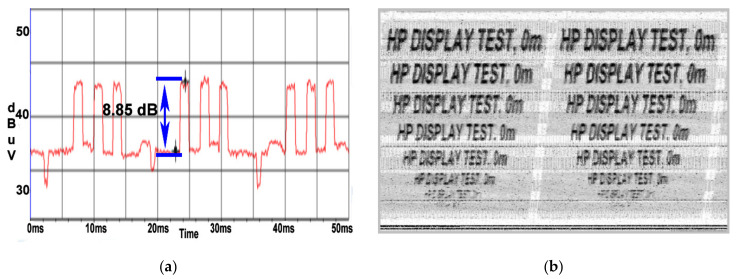
HP display: (**a**) SNR measured at a distance of 0 m; (**b**) image recovered at a distance of 0 m.

Compared to the AOC, the HP display has a lower SNR, of 8.8 dB, as illustrated in Figure 15a, when directly connected to the LISN and the image was successfully recovered, despite the presence of the perturbance occurred through the power line with a higher amplitude than the CE.

In this case, in order to reduce the effect of the additional noise existing on the receiving frequency, a special function of the TEMPEST specialized command and control system called “Image Averaging” has been utilized. This function works by overlapping more video frames which have the same rate of repetition in order to decrease the impact of a parasite signal present on the same frequency with the CE.

**Figure 16:**
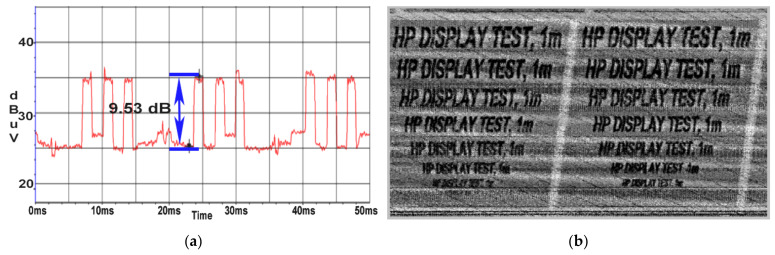
HP display: (**a**) SNR measured at a distance of 1 m; (**b**) image recovered at a distance of 1 m.

The value of the SNR surprisingly increased to 9.5 dB at a distance of 1 m, measured in Figure 16a, thus resulting in a more qualitative image reconstruction down to a font of 22 seen in Figure 16b. The amplitude of the targeted signal became comparable with the amplitude of the parasite signal caused by the EUT’s power supply.

**Figure 17:**
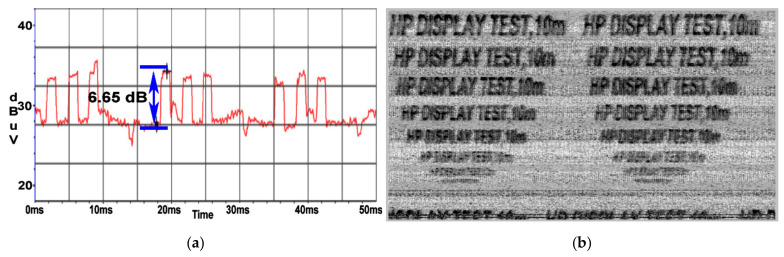
HP display: (**a**) SNR measured at a distance of 10 m; (**b**) Image recovered at a distance of 10 m.

In Figure 16b, it can be seen that the restored image containing text is overlaid on a series of oblique lines. These oblique lines are parallel and equidistant which indicates that they correspond to a periodic signal. These lines come from the periodic signal reported above, in Figure 6b, which has a period of 20 ms which is equivalent to the frequency of 50 Hz. As it can be observed in Figure 17a, at a distance of 10 m, the SNR decreased to a value of 6.6 dB and the recovered image degraded in quality, being able to distinguish the text until a font of 36. It should be noted that in Figure 17b the oblique lines shown in Figure 16b are no longer visible. By inserting the 10 m extension cable an additional attenuation is introduced which also attenuates the level of the disturbance noise generating these lines.

**Figure 18:**
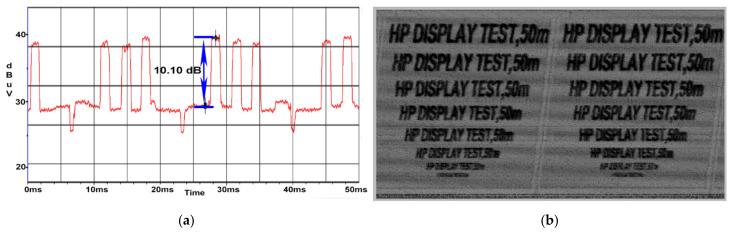
HP display: (**a**) SNR measured at a distance of 50 m when the cable reel was wound; (**b**) image recovered at a distance of 50 m when the cable reel was wound.

The phenomena emphasized in Figure 13a,b persists in the case of the HP display unit with a measured SNR of 10.1 dB as shown in Figure 18a,b, the coil effect of the wire wound on the cable reel acting in the same way despite the CE propagation on a different frequency.

**Figure 19:**
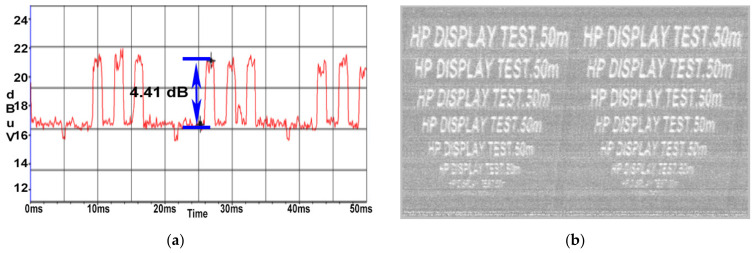
HP display: (**a**) SNR measured at a distance of 50 m; (**b**) image recovered at a distance of 50 m.

While the power cord has been unwound from the cable reel it has been received the lowest value having a SNR of 4.4 dB and the less intelligible image has been recovered as illustrated in Figure 19a,b. We can conclude that the propagation of the CE is strongly influenced by the distance of the cable.

In all cases, for both displays tested, text with size 12 and 18 are not intelligible, and therefore we can consider that this information could not be recovered

In Table 1, we have synthesized the values of the SNRs evaluated for both EUTs at all measurement distances realized during our research.

## 5. Countermeasures

In Section 3, we presented the frequency ranges in which compromised emissions were received on the power supply line of the tested equipment, respectively 460–510 MHz for the AOC display device and 250–300 MHz for the one produced by HP. The reported phenomenon is not a singular one and the electromagnetic disturbance propagates in other frequency ranges as well but we have selected the most representative frequency ranges for our test. In addition, in Section 4, we illustrated results according to which the maximum recorded SNR was received on the 274 MHz frequency for the HP monitor, at values of 6.6 dB for a distance (electric cable length) of 10 m and 4.4 dB for 50 m respectively, as shown in Table 1.

Based on the results obtained during our research, we primarily recommend the use, whenever possible, portable electronic devices that can operate for a limited period of time (tens of minutes or hours) without connection to the power supply source such as smart phones, multifunctional tablets or laptops (portable computers).

Unfortunately, this solution cannot be applied in any situation of daily reality, for example in the case of video projectors or personal computers that do not support battery power supply. In this situation, uninterruptible power supply (UPS) devices can be used, but without being connected to the power supply mains for a specific period of time, e.g., between 30 min and two hours. In this situation, the sound notification of this equipment, which informs the user of the interruption of the power supply, must be deactivated in order to allow the work in their proximity, if this function is allowed by the targeted UPS equipment. In this way, the electromagnetic disturbances generated by the electronic equipment cannot propagate on the power supply line because they are not connected to it.

There is also a speculation in the scientific community that UPS devices filter out the compromising disturbances generated by electronic equipment. Unfortunately, we cannot confirm this information, and during our entire TEMPEST equipment evaluation activity, this claim has proven to be false. From the tests carried out thus far, it has been observed that UPS devices generate additional noise on the power line which partially covers or masks the electromagnetic disturbances transmitted by the electronic equipment on the power line and we intend to present some illustrative examples in our future research to demonstrate this phenomenon.

Basically, the effect of UPS-type devices can be compared in some way to the effect of electromagnetic jamming equipment for the compromising emissions radiated into free space by electronic equipment. Only unlike jamming equipment, the electromagnetic disturbances additionally injected by UPS equipment on the power line cannot be controlled in amplitude and frequency range. The role of UPS devices is to protect the information being processed by IT&C electronic equipment in the event of power supply breakdown and even to ensure energy independence for a certain period of time. The existence of these equipment is also imposed for the protection of the hardware components of the electronic equipment such as the hard disk drives (HDD) of the personal computers.

It would have been desirable and cost effective if these power supply devices could also solve the problem reported in our research. Unfortunately, the electromagnetic noise induced by UPS equipment in the power supply network is usually observed up to a maximum frequency of 300 MHz and does not mask the compromising disturbances analysed in this paper on any frequency on which it was detected. Thus, we can say that UPS devices have an uncontrolled, unpredictable and insufficient effect from this point of view.

Usually, for any space or room in a building, there are at least two distinct electrical circuits: an electrical circuit that provides power to the electrical lighting of that space and at least one electrical circuit to power the electrical outlets installed in that space. In order to filter out the electromagnetic disturbances injected by the electronic equipment in the power supply network, it is recommended to install specialized EMI filters on one of the electrical circuits that supply the electrical sockets in the targeted space or room. It is not necessary to purchase high-performance filters such as those used in the construction of specialized shielded enclosures (such as in the medical, electromagnetic compatibility or TEMPEST field) but some less efficient. In the case of specialized shielded enclosures (EMC or TEMPEST), the installation of EMI filters is performed by hermetically sealing (360 degrees) on the outside of the electromagnetic screen by using specialized EMI gaskets. Exceptions to this rule are the shielded enclosures made by applying shielding materials (sheet, wallpaper, paint) on the walls of a room. In this situation, EMI filters are applied indoors as there is no other possibility to be connected to the electromagnetic shield. The installation of EMI filters is imposed by the phenomenon of re-radiation of electromagnetic energy or electromagnetic induction between electrical cables or between electrical cables and data cables.

When purchasing such devices, we recommend, as technical specifications, an insertion loss of at least 20 dB in the frequency range 10 MHz–1GHz. The value of 20 dB was established based on the signal-to-noise ratio (SNR) recorded during our research. For electronic equipment that generates compromising electromagnetic disturbances with an SNR greater than 20 dB, these filters will no longer be effective.

EMI filters that are used in specialized EMC or TEMPEST shielded enclosures must have a minimum insertion loss of 60–100 dB and the frequency range is much wider. These specifications together with the filter power consumption (maximum current allowed at rated voltage) reflect the purchase price of the EMI filter. The maximum consumption variants for these filters are generally for currents of 4, 6, 10, 16, 32 amps or even higher.

If it is necessary to apply such a filter on the entire socket circuit, we consider that the most suitable value is 16 amps but if we need to filter an isolated power line (which supplies for example a personal computer and a video projector) then it would be more recommended the value of 4 amps. In general, it is good to provide an additional consumption of 20–30% to avoid the possibility of burning the EMI filters. All these aspects related to energy consumption are simple and intuitive, but our readers should not neglect these aspects if they intend to make such purchases. In the case of an office space, EMI filters will be installed along the power cables (electrical extension with a minimum length of 3 m) and in this situation it is necessary to use shielded electrical cables [28,29,30,31] whose screen must be hermetically connected (360 degrees) to the EMI filter housing.

For companies that care about the confidentiality of business information, we recommend the application of security measures for a single room of reasonable size to allow the participation in confidential meetings of a minimum of 10 people, such as meeting rooms. By security measures applied to the physical space, we mean ensuring minimum protection measures against unwanted electromagnetic emissions such as the application of EMI filters for a small number of power outlets and the application of electromagnetic curtains or drapes. Studies on the shielding efficiency of electromagnetic curtains and drapes will be the subject of further future research.

Another protection measure against compromising disturbances analysed in this research and which involves cost efficiency is the application of clamp-on ferrite beads (or rings) on the power cable of the targeted electronic equipment. Ferrite beads are passive electronic components that can suppress high frequency signals on a power supply line. Normally, these are placed around a pair of power or ground lines near the termination of the cable (near the electronic enclosure), but they may also be installed at both ends if the cable connects two separate enclosures containing RF sources. By applying ferrite beads, the magnetic core around a conductor induces an inverse electromagnetic field (EMF) in the presence of a high frequency signal, essentially attenuating the frequency response of the ferrite. This application of ferrite beads ensures suppression or elimination of conducted electromagnetic interference (EMI) on the power line by attenuating high frequency EMI. Ferrite beads means a ceramic material made of iron oxide combined with certain other metal oxides, commonly used for inductive components. Ferrites can be an effective tool for eliminating radio frequency (RF) interference between electronic, information technology or telecommunications systems and to use them effectively, we need to understand them. Ferrites are ceramics formed from various metal oxides (metal oxides + iron oxide) in order to obtain a high magnetic permeability. Oxides of iron, magnesium, manganese and zinc are the most commonly used oxides, resulting in manganese–zinc (MnZn), nickel–zinc (NiZn) and magnesium–zinc (MgZn) alloys. The most common ferrites are NiZn and MgZn. Nickel–zinc ferrite beads work best for low power, high inductance circuits operating in the 500 KHz to 100 MHz frequency range because of low permeability, high volume resistivity, good temperature stability, and high Q factors. It is recommended to use them in the frequency range between 2 and 500 MHz. Manganese-zinc ferrite beads have high permeabilities that range above 800 μ, low volume resistivity, and low Q factors and is recommended to use them in the frequency range between 2 to 250 MHz. Manufacturers vary the chemical composition (mixture or proportion) and ferrite sizes to achieve the desired electrical and electromagnetic performance characteristics. However, we are only users, and we are not ferrite manufacturers; thus, it is not our problem how to obtain ferrites; we only need to know how to choose them.

Ferrite rings or ferrites beads works as a low-pass filter that allows only low-frequency signals to pass through a circuit and eliminates high-frequency noise. Ferrite beads do not act as a wideband low-pass filter as they can only help attenuate a specific range of frequencies. In general, ferrites should be placed as close as possible to the source of the noise, as noise can become trapped in unfiltered traces and cables. Ferrite beads are categorized by three response regions: inductive, resistive, and capacitive. To reduce high frequency noise, the bead must be in the resistive region which is especially desirable for electromagnetic interference (EMI) filtering applications. Ferrite beads act as a resistor (whose value depends on the frequency), which prevents high-frequency noise from passing through and dissipates it as heat. The resistive region occurs after the bead crossover frequency (reactance X = resistance R) and up to the point where the bead becomes capacitive. This capacitive point occurs at the frequency where the absolute value of capacitive reactance is equivalent to R. If more than one loop is made on the ferrite ring or the electric cable is passed through the ferrite bead two or three times, a substantial increase of ferrite impedance and a slight decrease of resonant frequency will be obtained.

However, for EMI filtering purposes, it is desirable that the ferrite bead has a high impedance over a wide frequency range. Their impedance is related to the material used (material permeability), the size of the ferrite bead, the number of windings and the construction of the winding. Similar to all inductors, the impedance of a ferrite bead is roughly proportional under resonance to the square of the number of turns passing through the core. The resonance of the ferrite is closely related to its dimensions due to the propagation velocity in the ferrite and the standing waves that are set in the cross-sectional dimensions of the core. In general, for any ferrite, the smaller the core, the higher the frequency of this resonance will be, and the resonant frequency will double if the core size is halved. Alternating current (AC) resistance is the peak impedance where the ferrite bead appears to be purely resistive while the direct current (DC) resistance is acquired from the manufacturer data sheet. For the frequencies where the component resistance is the majority, ferrite beads show their property to absorb noise by converting it to heat. Since the DC resistance of an inductor can generally cause power losses but also heat generation, it is desirable that the DC resistance is low when using an inductor for a power supply. For a low-voltage power supply with low allowable voltage ripple, a component with low DC resistance should be chosen to avoid large power losses within the desired signal source.

Selecting the right ferrite bead for power applications requires careful consideration not only of the filter bandwidth, but also of the impedance characteristics of the bead with respect to DC bias current. In most cases, manufacturers only specify the impedance of the bead at 100 MHz and publish data sheets with frequency response curves at zero DC bias current. As the DC bias current increases, the core material begins to saturate, which significantly reduces the inductance (permeability) of the ferrite bead. The degree of inductance saturation differs depending on the material used for the core of the component. For effective power supply noise filtering we should use ferrite beads at about 20% of their rated DC current. In addition, the effect of DC bias current can be observed in the reduction of impedance values over frequency, which in turn reduces the effectiveness of the ferrite bead and its ability to remove EMI.

Standard ferrite beads can be acquired from specialized manufacturers such as Wurth Elektronik, TDK, Laird, Murata, Epcos, Micrometals, Coilcraft and more others. This solution does not always eliminate the problem but can reduce the risk of interception of sensitive information by reducing these disturbances.

**Figure 20:**
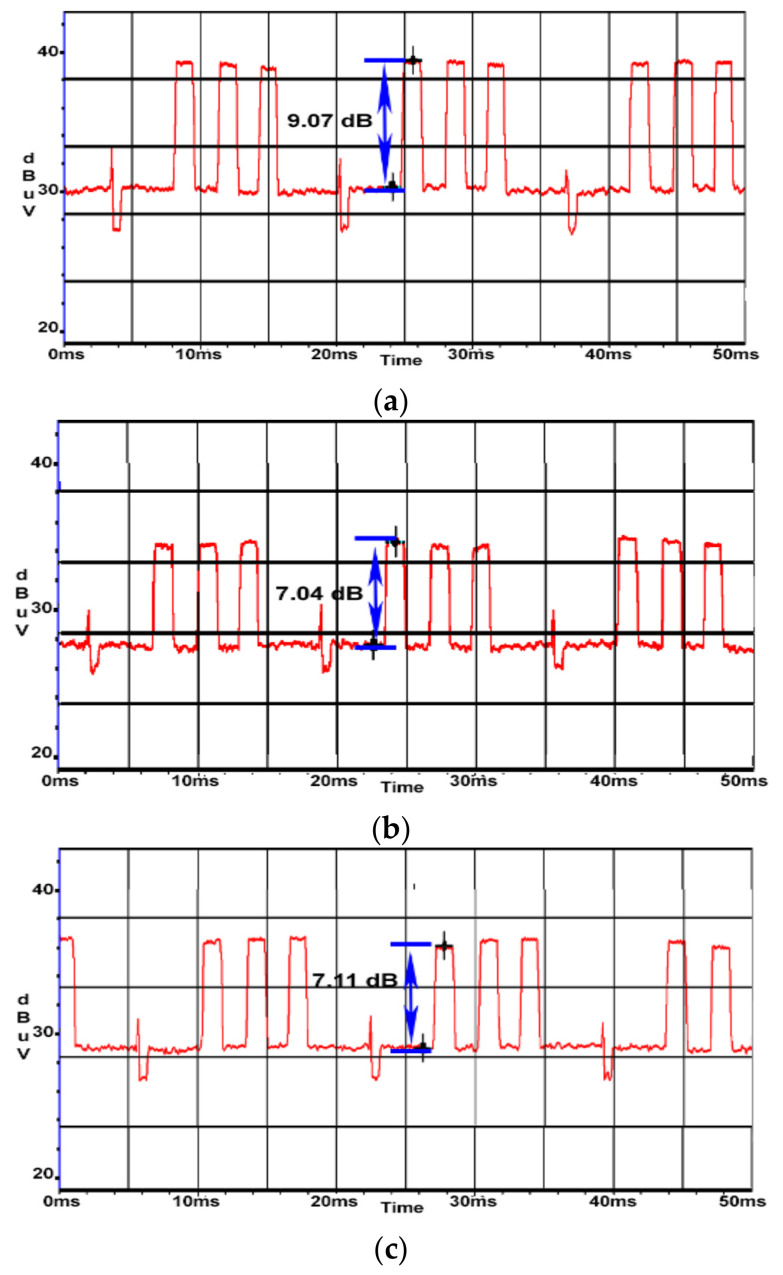
AOC display: (**a**) SNR measured for display’s power cable with no ferrites (SNR = 9.0 dB); (**b**) SNR measured for display’s power cable with ferrite TDK SEIWA 18 mm ferrite at the IEC C13 end (SNR = 7.0 dB); (**c**) SNR measured for display’s power cable with ferrite TDK SEIWA 18 mm ferrite at the schuko end (SNR = 7.1 dB).

In support of this statement, the results shown in the figures below are illustrated by using some of the ferrites available in the laboratory at the time of testing. The utilised ferrite beads have a diameter of 18 mm which allows them to be applied by double wrapping the power cable. Thus, Figure 20a shows the SNR recorded without the application of the ferrite beads while Figure 20b,c shows the SNR recorded after the application of the ferrite at one end and respectively the other of the monitor power cord.

**Figure 21:**
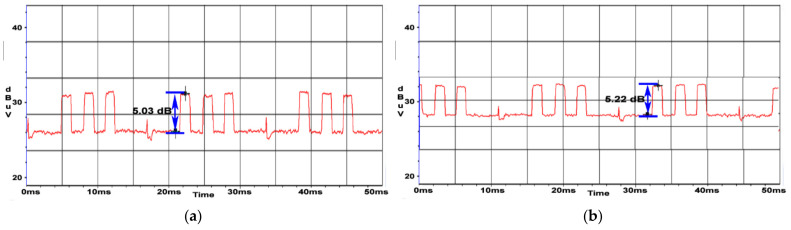
AOC display: (**a**) SNR measured for display’s power cable with TDK SEIWA 18 mm ferrites at both ends (SNR = 5.0 dB); (**b**) SNR measured for display’s power cable with FAIR-RITE VO 18 mm ferrites at both ends (SNR = 5.2 dB).

An attenuation of about 2 dB was obtained in both cases. By applying the ferrite beads to both ends of the power cord, a reduction in SNR of about 4 dB was obtained. In Figure 21a, TDK SEIWA ferrite rings were used, while in Figure 21b, FAIR-RITE VO products were used.

There are studies that provide the calculation of geometric dimensions for these ferrite rings [32,33,34,35], but a good starting point for people not specialized in this field are the ferrites rings applied on the video cables for the VGA interface.

## 6. Conclusions

Our research presents a new approach for the TEMPEST analysis perspective by involving long distance SNR measurements and image reconstruction. The linear behaviour of the electromagnetic propagation phenomena of the electromagnetic disturbances generated unintentionally by the two analysed displays was observed. An exception to this statement was found in the case of the use of the 50 m extension cable wrapped on the roller. We also carried out tests in this situation to highlight the electromagnetic induction phenomenon that occurs between the power cable loops.

Unlike the propagation of electromagnetic waves in free space, in the case of electromagnetic propagation along a conductor, as presented in this research, such as in the case of electrical conductors, no significant attenuation was recorded. This aspect underlines the vulnerability of the information processed by electronic equipment to electromagnetic disturbances generated by this equipment and transmitted through the power line. As is known, the propagation of electromagnetic radiation in free space, given by the Friis formula [36], gives us a good estimate, accepted by the whole scientific community. According to this formula, the difference between the radiated propagation of electromagnetic waves in free space (free space loss) at a distance of 1 m and at a distance of 50 m is no longer frequency dependent and is approximately 34 dB (33.98 dB). The conducted propagation of electromagnetic disturbances on the power line is, however, not as scientifically well established and therefore is not easy to estimate. According to the results recorded and presented in this paper, the difference between the conducted propagation of electromagnetic waves along a power cable (consisting of 3 multi-wire electrical copper cables with a diameter of 2.5 mm, valid for all the extension cables we used) with a length of 1 and 50 m was 6.3 dB for the AOC monitor (at the reception frequency of 484 MHz) and 5.1 dB for the HP monitor (at the reception frequency of 274 MHz).

Taking into account that the same measurement configuration was used for both monitors as well as the same extension electrical cables, we can conclude that the conducted propagation of electromagnetic waves is frequency dependent as opposed to the radiated propagation. We can also observe that the attenuation difference of conducted propagated electromagnetic waves is about seven times lower than that of radiated propagated ones, and we can thus state that they are much more dangerous in terms of long-distance propagation and vulnerabilities related to the confidentiality of information processed by electronic equipment.

In Section 5, countermeasures to protect against these phenomena have been presented, including the financial aspects of their application. In the case of the experiments presented in Section 5, an attenuation of 4 dB was recorded when applying ferrite beads to both ends of the power cable of the tested equipment. For these tests, ferrites were not purchased on the basis of previous calculations but utilised those from the TEMPEST laboratory where the tests were carried out. This value proved to be sufficient, according to our research, to attenuate electromagnetic disturbances propagated at a distance of 50 m but may also obstruct the detection and restoration of the video signal for shorter distances.

## Figures and Tables

**Table 1:** SNR measured for both displays at several distances.

Type of Display	Distance (m)	SNR (dB)
AOC	0	9.7
	1	9.3
	10	3.5
	50 (wound cable)	9.0
	50	3.1
HP	0	8.8
	1	9.5
	10	6.6
	50 (wound cable)	10.1
	50	4.4

## References

[1] European Committee for Electrotechnical Standardization (CENELEC) (2008). Limits and Methods of Measurement of Radio Interference Characteristics of Information Technology Equipment.

[2] International Electrotechnical Commission (IEC) (2019). Specification for Radio Disturbance and Immunity Measuring Apparatus and Methods—CISPR 16-2-3.

[3] International Electrotechnical Commission (IEC) (2010). Information Technology Equipment—Radio Disturbance Characteristics―Limits and Methods of Measurement, CISPR 22.

[4] US Department of Defence Requirements for the Control of Electromagnetic Interference Characteristics of Subsystems and Equipment. MIL-STD-461G. 11 December 2015. https://govtribe.com/file/government-file/attachment-2-mil-std-461g-dot-pdf.

[5] Council of the European Union, The General Secretariat TEMPEST and EMS Policy. https://www.ncsc.gov.uk/information/tempest-and-ems-policy.

[6] Council of the European Union, The General Secretariat Information Assurance Security Guidelines on Accreditation of EU TEMPEST Companies. https://data.consilium.europa.eu/doc/document/ST-7887-2019-INIT/en/pdf.

[7] NATO Military Committee Communication and Information Systems Security and Evaluation Agency (SECAN), Supreme Headquarters Allied Powers Europe (SHAPE) (2009). NATO Standard (2009) SDIP-27/2: NATO TEMPEST Requirements and Evaluation Procedures.

[8] General Secretariat of the Council of the European Union (GSC) (2013). EU Standard (2013) IASG 7–03: Information Assurance Security Guidelines on EU TEMPEST Requirements and Evaluation Procedures.

[9] Van Eck W. (1985). Electromagnetic radiation from video display units: An eavesdropping risk?. Comput. Secur..

[10] Kuhn M.G., Anderson R.J. (1998). Soft Tempest: Hidden Data Transmission Using Electromagnetic Emanations. International Work-Shop on Information Hiding.

[11] Kuhn M.G. Compromising Emanations: Eavesdropping Risks of Computer Displays. https://www.cl.cam.ac.uk/techreports/UCAM-CL-TR-577.pdf.

[12] Kuhn M.G. (2004). Electromagnetic eavesdropping risks of flat-panel displays. Proceedings of the 4th Workshop Privacy Enhancement Technology.

[13] Nowosielski L., Przesmycki R., Nowosielski M. Compromising Emanations from VGA and DVI Interface. Proceedings of the 37th Progress in Electromagnetics Research Symposium (PIERS).

[14] Przesmycki R. High Definition Multimedia Interface in the Process of Electromagnetic Infiltration. Proceedings of the 36th Progress in Electromagnetics Research Symposium.

[15] Kubiak I. (2016). Video signal level (colour intensity) and effectiveness of electromagnetic infiltration. Bull. Pol. Acad. Sci..

[16] Kubiak I. (2019). Influence of the method of colors on levels of electromagnetic emissions from video standards. IEEE Trans. Electromagn. Compat..

[17] Kubiak I. (2017). LED printers and safe fonts as effective protection against the formation of unwanted emission. Turk. J. Electr. Eng. Comput. Sci..

[18] Kubiak I., Boitan A., Halunga S. (2020). Assessing the Security of TEMPEST Fonts against Electromagnetic Eavesdropping by Using Different Specialized Receivers. Appl. Sci..

[19] Boitan A., Kubiak I., Halunga S., Przybysz A., Stańczak A. (2020). Method of Colors and Secure Fonts Used for Source Shaping of Valuable Emissions from Projector in Electromagnetic Eavesdropping Process. Symmetry.

[20] Kubiak I., Loughry J. (2020). Special Computer Fonts in Electromagnetic Safety of Digital Graphic Standards.

[21] Suzuki Y., Masugi M., Tajima K., Yamane H., Countermeasures to Prevent Eavesdropping on Unintentional Emanations from Personal Computers NTT Energy and Environment Systems Laboratories Musashino-shi, 180-8585 Japan; October 2008; Volume 6, No. 10. https://www.ntt-review.jp/archive/ntttechnical.php?contents=ntr200810sf2.html.

[22] Przybysz A., Grzesiak K., Kubiak I. (2021). Electromagnetic Safety of Remote Communication Devices—Videoconference. Symmetry.

[23] Liu Z., Samwel N., Weissbart L., Zhao Z., Lauret D., Batina L., Larson M. (2020). Screen Gleaning: A Screen Reading TEMPEST Attack on Mobile Devices Exploiting an Electromagnetic Side Channel. arXiv.

[24] Efendioglu H.S., Asik U., Karadeniz C. Identification of Computer Displays Through Their Electromagnetic Emissions Using Support Vector Machines. In Proceedings of the 2020 International Conference on INnovations in Intelligent SysTems and Applications (INISTA).

[25] Nassi B., Pirutin Y., Galor T.C., Elovici Y., Zadov B. (2021). Glowworm Attack: Optical TEMPEST Sound Recovery via a Device’s Power Indicator LED.

[26] TEMPEST AMN (LISN) TEMP 8400, Schwarzbeck Mess—Elektronik OHG. http://schwarzbeck.de/Datenblatt/k8400.pdf.

[27] Test Receiver Rohde&Schwarz (R&S) FSET7, R&S FSET22. https://www.rosenkranz-elektronik.com/userUpload/pdf/fset7%2022%20-z2%20-z22%20datasheet.pdf.

[28] Shielded In-Wall Cable, Aaronia AG (aaronia-shop.com). https://aaronia-shop.com/products/shielding-materials/shielded-cables/shielded-in-wall-cable-50m-2.

[29] Shielded Power Cables, Cervinor, S.A. https://www.cervi.es/Documentos/FX_994.pdf.

[30] Shielded Power Cables, HELUKABEL. https://www.tme.eu/Document/da17018489b0e64522074539ceedfd25/B%20025%20EDV-PiMF-CY.pdf.

[31] Shielded Power Cables, Alpha Wire. https://www.tme.eu/Document/9a8d29cca3a9f896cc18289f34868790/1737C.pdf.

[32] Ghosh M.K., Gao Y., Dozono H., Muramatsu K., Guan W., Yuan J., Tian C., Chen B. (2019). Numerical Modelling of Magnetic Characteristics of Ferrite Core Taking Account of Both Eddy Current and Displacement Current. Heliyon.

[33] Furuya A., Uehara Y., Shimizu K., Fujisaki J., Ataka T., Tanaka T., Oshima H. (2017). Magnetic field analysis for dimensional resonance in Mn-Zn ferrite toroidal core and comparison with permeability measurement. IEEE Trans. Magn..

[34] Suarez A., Victoria J., Torres J., Martinez P.A., Alcarria A., Perez J., Garcia-Olcina R., Soret J., Muetsch S., Gerfer A. (2020). Performance Study of Split Ferrite Cores Designed for EMI Suppression on Cables. Electronics.

[35] The International Magnetics Association (IMA) (2011). IMA-STD-100. Soft Ferrite Cores, A User’s Guide. https://assets.noviams.com/novi-file-uploads/tta/pdfs-and-documents/Soft_Ferrite_Cores_User.

[36] (2019). International Telecommunication Union—Radiocommunication Sector, Calculation of Free-Space Attenuation, ITU-R P. 525.

